# Molecularly Targeted Small Molecule Inhibitor Therapy for Pediatric Acute Lymphoblastic Leukemia: A Comprehensive Review of Clinical Trials

**DOI:** 10.3390/cancers17203322

**Published:** 2025-10-15

**Authors:** Nicolò Peccatori, Erica Brivio, Andrej Lissat, Francisco Bautista Sirvent, Elisabeth Salzer, Andrea Biondi, Grazia Fazio, Carmelo Rizzari, Sarah K. Tasian, Christian Michel Zwaan

**Affiliations:** 1 Tettamanti Center, Fondazione IRCCS San Gerardo dei Tintori, 20900 Monza, Italy; 2 School of Medicine and Surgery, University of Milano-Bicocca, 20126 Milan, Italy; 3 Pediatric Hematology-Oncology Unit, Department of Pediatrics, Fondazione IRCCS San Gerardo dei Tintori, 20900 Monza, Italy; 4 Princess Máxima Center for Pediatric Oncology, 3584 CS Utrecht, The Netherlands; 5 Charité Universitätsmedizin, 10117 Berlin, Germany; 6 Willem Alexander Children’s Hospital, Leiden University Medical Center, 2333 ZA Leiden, The Netherlands; 7 Division of Oncology and Center for Childhood Cancer Research, Children’s Hospital of Philadelphia, Philadelphia, PA 19104, USA; 8 Department of Pediatrics and Abramson Cancer Center, Philadelphia, PA 19104, USA; 9 Department of Pediatric Oncology, Erasmus MC-Sophia Children’s Hospital, 3015 GD Rotterdam, The Netherlands

**Keywords:** acute lymphoblastic leukemia, clinical trials, inhibitor, pediatric, precision medicine, relapsed/refractory, targeted therapy

## Abstract

In the past decades, significant advancements in the biological and genetic characterization of acute leukemias and optimization of risk-adapted multi-agent treatment protocols have dramatically improved cure rates and quality of life for children with acute lymphoblastic leukemia (ALL). Despite these optimal results, patients with relapsed or chemotherapy-refractory (R/R) disease or with high-risk genetic features still face unsatisfactory outcomes. Further intensification of conventional chemotherapy has reached its limits in achieving the desired efficacy without undue side effects, necessitating innovative approaches to improve cure rates while continuing to minimize the toxicities associated with chemotherapy and hematopoietic stem cell transplantation. In the era of precision medicine, two key therapeutic strategies have emerged in hemato-oncology: molecularly targeted therapies and immunotherapies. Antibody-based and cellular immunotherapies have undoubtedly reshaped the landscape of childhood ALL treatment and have significant potential to play leading roles in current and future frontline regimens; these important therapies are well delineated in recent reviews. Molecularly targeted small molecule inhibitor therapies remain a cornerstone of precision medicine, supported by recent advancements in next-generation sequencing, which have enabled the application of transcriptomic and genomic profiling data to risk stratification and therapy optimization. Clinical trials for children with ALL have been instrumental in refining therapies and improving outcomes, a paradigm that remains critical as treatment strategies become increasingly complex. This comprehensive review focuses upon molecularly targeted therapy approaches for childhood ALL and aims to summarize findings from completed clinical trials to highlight the current landscape of ongoing and upcoming trials and to provide insights into future directions for the precision-driven optimization of pediatric B-ALL and T-ALL treatment.

## Simple Summary

Acute lymphoblastic leukemia (ALL) is the most frequent cancer of childhood. Although most children are cured with modern frontline chemoimmunotherapy regimens, those with high-risk genetics and/or relapsed/refractory diseases still face poor outcomes. There is a growing need for more precise and less toxic treatment options than traditional chemotherapy. This comprehensive review focuses upon the investigation of biologically relevant small molecule inhibitors for genetic subtypes of childhood B-ALL or T-ALL. We search a large international clinical trial database (clinicaltrials.gov) to identify studies that tested molecularly targeted drugs in children with ALL. By reviewing the results of all completed trials and presenting an overview of ongoing and upcoming studies, we aim to provide a clear picture of how these therapies have been developed so far and discuss the current direction of precision medicine in pediatric ALL.

## 1. Introduction

The cure rates and the quality of life of children with acute lymphoblastic leukemia (ALL) have significantly improved in recent decades due to extraordinary scientific advancements in the characterization of the disease from a biological and genetic perspective and the optimization of risk-stratified multi-agent treatment protocols within the framework of international studies [1,2,3]. The conduction of pediatric-specific ALL therapeutic clinical trials has been a pivotal factor in the dramatic improvement of outcomes observed over the recent decades, offering a structured, evidence-based approach to refining multi-agent chemotherapy protocols. The long-term disease-free survival of children with B-acute lymphoblastic leukemia (B-ALL) now exceeds 90% [3], while outcomes for those with T-ALL are approximately 85% [4]. Despite these excellent results, patients with high-risk genetic features and those who experience a relapse or who are refractory to frontline therapy still have a poorer prognosis (long-term survival ∼50% after first relapse) [5]. While treatment options have also improved in the relapsed/refractory setting, patients with B-ALL continue to have more effective therapeutic options than those with T-ALL. The outcomes of children with relapsed/refractory T-ALL are extremely poor (survival rate ∼30% for first relapse T-ALL [6]) and they require successful allogeneic hematopoietic stem cell transplantation (HSCT) for long-term survival [5,7]. The intensification of conventional treatment based on the use of chemotherapeutic drugs has further reached its maximal limit with minimal gains in improving outcomes for patients with chemo-refractory disease. Innovative treatment approaches based upon disease biology or cell surface antigens are essential to improve the chances of being cured and potentially also to decrease chemotherapy- and HSCT-associated toxicities [8]. In the era of precision medicine in hemato-oncology, two main categories of innovative therapeutic approaches can be distinguished: immunotherapy and molecularly targeted therapy [9,10]. Successful immunotherapeutic modalities, including monoclonal antibodies, antibody–drug conjugates, bispecific antibodies, and chimeric antigen receptor T-cell therapy (CAR-T), have clearly emerged as extremely effective therapeutic approaches, which have reshaped the scenario for treatment options in relapsed/refractory ALL and more recently also in the frontline setting, and which are reviewed elsewhere [11,12,13,14,15]. As an example, the remarkable results of the recent COG AALL1731 trial [16], which clearly demonstrated the superiority of a blinatumomab-based therapy (two non-sequential blinatumomab cycles added to a standard multi-agent chemotherapy regimen) in newly diagnosed children with Philadelphia chromosome-negative NCI standard risk B-ALL, represent a paradigmatic proof of concept.

**Figure 1:**
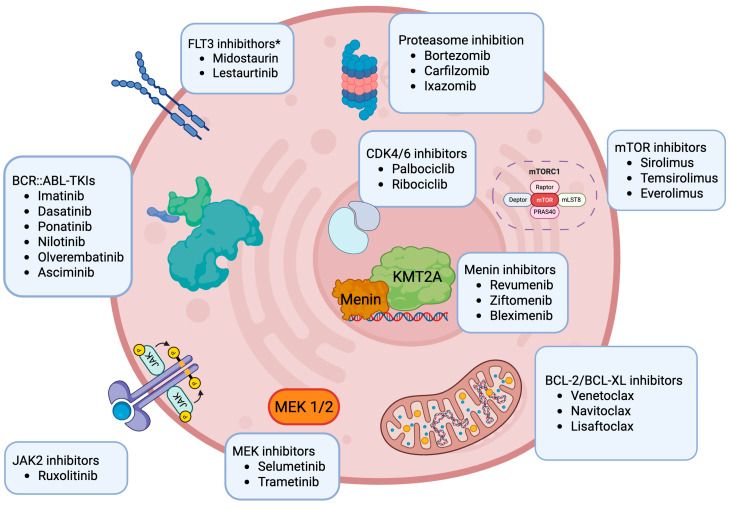
Overview of current molecularly targeted treatment in development in pediatric acute lymphoblastic leukemia. Created in https://BioRender.com. Among the drugs listed in Figure 1, only imatinib and dasatinib are currently approved by both the FDA/EMA for pediatric and adult ALL. Revumenib is FDA-approved for R/R KMT2Ar leukemias in patients one year and older. Ponatinib is approved in adults but not in children. The remaining drugs do not yet have a formal approval for ALL. * Though FLT3 inhibitors have been tested in ALL pediatric clinical trials in the past, these agents are not routinely added to chemotherapy for pediatric ALL patients at this time.

Molecularly targeted therapeutic approaches also represent a fundamental paradigm of successful precision medicine in ALL. For instance, the addition of tyrosine kinase inhibitors (TKIs) to chemotherapy has markedly improved the clinical outcomes for children with Philadelphia chromosome-positive (Ph+) ALL and is now standard in care, and, additionally, clinical trial efforts are ongoing to credit TKI-based therapy for patients with *BCR*::*ABL1*-like/Philadelphia-like (Ph-like) ALL [17,18]. The integration of next-generation sequencing (NGS) via transcriptome and genome profiling into the routine diagnostic algorithm for pediatric ALL has further allowed for the identification of new prognostic markers and optimized risk stratification, as well as guiding the selection of appropriately targeted therapies in clinical trials and compassionate use programs, and for off-label applications [19]. Several novel small molecules targeting molecular aberrations or specific molecular pathways are accordingly under preclinical and/or early-phase clinical investigation in children with relapsed leukemias (Figure 1). These agents hold promise for future integration into molecularly guided treatment strategies aimed at optimizing ALL therapy [20]. This paradigm is even more critical in the current context, marked by increasing biological complexity and the rapidly expanding landscape of novel treatment strategies and combinations. At the same time, increasingly, small patient populations with specific high-risk leukemia genetic subtypes call for strong international collaboration to ensure the feasibility of clinical trials and the generation of robust data to further improve outcomes for children with ALL. In this review, we provide a comprehensive overview of the clinical trial investigations into molecularly targeted agents in children with ALL that summarizes and discusses the available results of completed trials and highlights the state-of-the-art of ongoing and registered soon-to-open trials.

## 2. Materials and Methods

The website https://clinicaltrials.gov was searched to identify clinical trials exploring molecularly targeted agents in pediatric ALL. Immunotherapeutic agents and cellular therapies, including CAR T-cells, were considered to be outside the scope of this review and thus were excluded from the search. Twenty-eight searches were conducted by one investigator (NP) on 15 May 2025, using the advanced search mode with the pre-defined term “acute lymphoblastic leukemia” in the “condition or disease” box. The age-range filter (child, 0–17) was applied. In addition, each search contained one of the following intervention/treatment terms which were pre-determined by the authors as the most relevant agents to be considered in this review: “imatinib”, “dasatinib”, “ponatinib”, “nilotinib”, “asciminib”, “olverembatinib”, “ruxolitinib”, “bortezomib”, “carfilzomib”, “ixazomib”, “venetoclax”, “navitoclax”, “lisaftoclax”, “selumetinib”, “trametinib”, “palbociclib”, “ribociclib”, “sirolimus”, “everolimus”, “temsirolimus”, “vorinostat”, “decitabine”, “azacitidine”, “midostaurin”, “lestaurtinib”, “revumenib”, “ziftomenib”, “bleximenib”. Eligibility criteria were defined a priori, and only interventional clinical trials for children diagnosed with ALL were searched in the period from 1 January 2000 to 1 May 2025. Studies with a status of unknown, withdrawn, terminated early or suspended before recruitment were excluded. The study statuses are reported as of 26 September 2025. The available results from the clinical trials included in this review were retrieved through the PubMed database and from available conference abstracts from the European Hematology Association (EHA) and the American Society of Hematology (ASH).

## 3. Results

**Figure 2:**
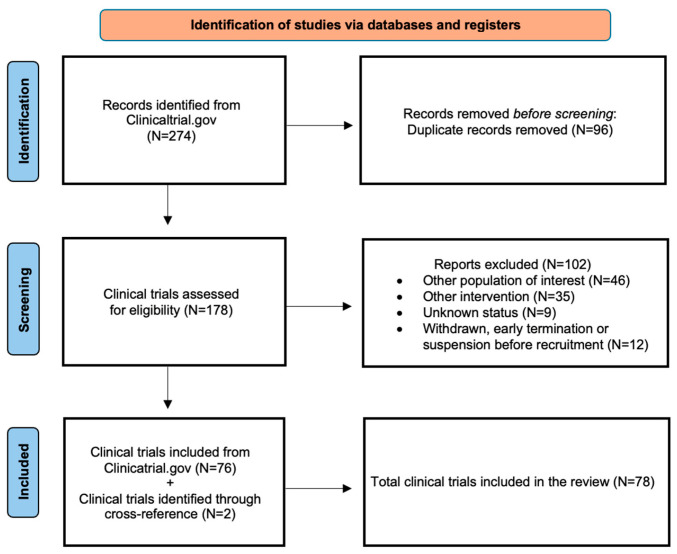
Flow diagram reporting the results of the searches performed on 15 May 2025 at https://clinicaltrials.gov.

The search strategy at clinicaltrials.gov yielded 274 clinical trials (Table S1). After removing the duplicate records which appeared in the various searches, as mentioned above, 178 studies were assessed for eligibility. Of those, 76 fulfilled the eligibility criteria (Figure 2). Two additional clinical trials were identified through cross-referencing [21,22] for a total number of 78 included clinical trials. The eligible studies were grouped in specific categories according to the molecular target or functional pathway and their results discussed as follows (Table 1). The toxicity profiles of small molecules considered in this review, organized by drug class, are summarized in Table 2.

### 3.1. BCR::ABL1-Directed Tyrosine Kinase Inhibitors

Ph+ ALL, defined by the canonical *BCR*::*ABL1* fusion from chromosomal translocation t(9;22) (q34;q11), represents the first paradigmatic example of the successful incorporation of a molecularly targeted therapy in the treatment of ALL [80]. This subtype of ALL, accounting for 25% of adults and 3–4% of pediatric patients with B-ALL, was historically considered the most unfavorable ALL subtype. However, starting in the early 2000s, the advent of TKIs directed against *BCR::ABL1* has revolutionized its treatment and significantly improved the prognosis of both adult and pediatric patients. In the last 20 years, the North America/Australia/New Zealand-based Children’s Oncology Group (COG) and the European multi-national Ph+ ALL group (EsPhALL) cooperative pediatric study groups have conducted several consecutive clinical trials in children with newly diagnosed Ph+ ALL (Table 1). These trials studied first- (imatinib) or second-generation (dasatinib) TKIs with different chemotherapy backbones and clearly demonstrated that this strategy significantly improved outcomes and avoids the need for HSCT in most children and adolescents with newly diagnosed Ph+ ALL [81]. More recently, such principles have also been applied to the Ph-like B-ALL subtype, which comprises 15–20% high-risk pediatric ALL cases and is characterized by a gene expression profile very similar to that of Ph+ ALL despite the lack of *BCR*::*ABL1* fusion. Ph-like ALL has proven to be quite genetically heterogeneous with >80 specific fusions identified to date, although the patients can largely be “binned” into one of two subgroups [81,82]. The ABL-class subtype involves fusions of *ABL1*, *ABL2*, *CSF1R*, or *PDGFRB*. Although genetically distinct from Ph+ ALL, ABL-class ALL has very similar biological, clinical, and prognostic characteristics and, importantly, appears to share with Ph+ ALL excellent sensitivity to TKIs in preclinical studies and clinical case series [18]. For this reason, pediatric patients with ABL-class Ph-like ALL are now also enrolled in international Ph+ ALL protocols for treatment with TKI-based therapies (Table 1). Besides imatinib and dasatinib, in recent years several new-generation *BCR::ABL1*-directed TKIs have been developed primarily for patients with chronic myeloid leukemia (CML), including nilotinib, bosutinib, ponatinib, and olverembatinib, and the specifically targeting ABL myristoyl pocket (STAMP) inhibitor asciminib. Despite testing to date being primarily in adult patients with CML or Ph+ ALL, data are now emerging in the pediatric-specific setting via early-phase clinical trials.

#### 3.1.1. Imatinib

Imatinib mesylate was the first TKI developed to treat Philadelphia chromosome-positive leukemias. It is FDA- and EMA-approved for treating CML and Ph+ ALL in adults and children. Imatinib was first tested in the pediatric population in the COG Phase I study (NCT00004932), which demonstrated the antileukemic activity and the favorable tolerance of daily oral single-agent imatinib at doses ranging from 260 to 570 mg/m^2^ in children and adolescents with R/R CML and R/R Ph+ ALL [23]. Based on these results, the first pilot study COG AALL0031 (NCT00022737) testing the efficacy of the combination of imatinib 340 mg/m^2^ and chemotherapy was carried out by the COG group in newly diagnosed Ph+ ALL pediatric patients between 2002 and 2006 [24]. This study tested differential schedules of imatinib (increasing number of chemotherapy blocks including imatinib) in 5 patient cohorts. It demonstrated that higher imatinib dosing in Cohort 5 (continuous exposure from consolidation 1 to maintenance) significantly improved the patient outcomes, with a 3-year event-free survival (EFS), which is more than twice that of historical controls (88 ± 11% vs. 35 ± 4%; *p* < 0.0001) [24]. No significant toxicities associated with adding imatinib to intensive chemotherapy were reported [24]. These superior results were further confirmed by long-term analysis, with the 5-year EFS of 70 ± 12% for the Cohort 5 patients treated with chemotherapy plus imatinib [83]. Notably, these outcomes were similar to those of patients treated with HSCT in first complete remission (CR1), with 5-year EFS of 65 ± 11% and 59 ± 15% (n = 13, *p* = 0.60) for related donor HSCT and unrelated donor HSCT, respectively [83].

At the same time as the COG AALL0031, the European EsPhALL group conducted a randomized trial (NCT00287105, EsPhALL 2004) that enrolled children from 2004 to 2009 with newly diagnosed Ph+ ALL to test whether outcomes were improved when incorporating discontinuous post-induction imatinib therapy (300 mg/m^2^) to an intensive Berlin–Frankfurt–Munster (BFM) chemotherapy backbone [25]. Patients stratified as good-risk were randomly assigned to receive chemotherapy alone vs. chemotherapy plus imatinib, while all poor-risk patients received chemotherapy plus imatinib. EsPhALL 2004 results confirmed that the addition of imatinib was beneficial in the treatment of children with Ph+ ALL, with intention-to-treat analysis showing for good-risk patients receiving imatinib a 4-year DFS of 73% vs. that of 62% for those who did not receive imatinib (*p* = 0.24). Importantly, in this study there was also a high rate (77%) of HSCT in CR1 in the good-risk patient cohort, meaning no conclusions can be drawn regarding the role of HSCT over TKI-containing multi-agent chemotherapy only, for good-risk Ph+ ALL patients. Despite the incorporation of TKI and the extensive use of HSCT, the outcomes for poor-risk patients remained unfavorable, with 4-year EFS of 53.3% [25]. Based upon the COG AALL0031 results published in 2009, which demonstrated the advantage of the earlier and continuous administration of imatinib, the EsPhALL 2004 study was amended in 2010 to test, in a single-arm clinical trial (EsPhALL 2010), continuously dosed imatinib from mid-induction (day 15) in association with the same intensive chemotherapy backbone used in EsPhALL 2004. Furthermore, the allocation of HSCT in CR1 was restricted to MRD-poorly-responding (end of induction 1B MRD > 5 × 10^−4^) or MRD-non-responding patients, reducing by half the HSCT rate for the overall cohort (38%) and particularly for the good-risk patients (32%). This amended trial (EsPhALL 2010) demonstrated that early and continuous use of imatinib concurrently with intensive chemotherapy can effectively replace HSCT in the majority of good-risk Ph+ ALL patients, resulting in a 5-year EFS of 62.7% [26]. Despite the integration of TKIs, both COG and EsPhALL chemotherapy backbones used to treat Ph+ ALL patients were highly intensive, resulting in high rates of treatment-related toxicities and mortality. Hence, these two major consortia designed a joint international collaborative randomized protocol (NCT03007147, EsPhALL 2017/COG AALL1631) to evaluate whether chemotherapy de-intensification in combination with imatinib could reduce treatment-related morbidity and mortality without adversely impacting the outcomes in standard-risk newly diagnosed Ph+ ALL patients. Conversely, for high-risk patients treated with imatinib in combination with intensive EsPhALL backbone and HSCT, this study aimed to describe the feasibility and outcomes of 1-year post-HSCT imatinib administration. The EsPhALL 2017/COG AALL1631 trial has recently completed its recruitment, representing to date the largest trial performed in pediatric Ph+ ALL, and its results will constitute the benchmark for novel clinical trials in the next years. As aforementioned, ABL-class fusion positive ALL is a high-risk ALL subtype associated with unfavorable outcomes when treated with conventional risk-adapted multi-agent chemotherapy [84], which is likely to benefit from the addition of TKIs such as imatinib and dasatinib as suggested by recent anecdotal evidence and retrospective studies [84,85,86]. Based on this consideration, the EsPhALL 2017/COG AALL1631 was amended in 2021 to also include a cohort of patients with ABL-class fusion positive ALL as eligible to participate in the chemotherapy backbone randomization. The ongoing ALLTogether1 trial (NCT04307576) also treats all patients with ABL-class ALL with non-random experimental intervention, with the addition of imatinib from the induction phase for patients < 25 years and from the consolidation phase for patients ≥ 25 years, with the aim to reduce the risk of disease recurrence.

#### 3.1.2. Dasatinib

Dasatinib is a second-generation TKI with dual ABL/SRC kinase inhibition activity approved by both the FDA and EMA for the treatment of pediatric patients with newly diagnosed Ph+ ALL in combination with chemotherapy. Compared to imatinib, dasatinib has higher in vitro inhibitory potency (>300-fold), and better central nervous system (CNS) penetration capacity and activity against some imatinib-resistant Ph+ clones [87]. The additional effect of dasatinib against SRC family kinases might contribute to higher efficacy in ALL [88]. The safety and efficacy profile of dasatinib (twice daily dosing of 50–110 mg/m^2^/dose) was firstly assessed in the pediatric population in a phase I study performed by COG (NCT00316953, CA180-038/COG-ADVL0516), which included patients with refractory solid tumors or imatinib-refractory Ph+ leukemias who showed drug disposition of and tolerability to dasatinib similar to that observed in adult patients, and found the maximum tolerated dose (MTD) to be 85 mg/m^2^/dose administered twice daily [28]. A subsequent European Phase 1 dose-escalation study of dasatinib (NCT00306202, CA180-018/ITCC-005), developed within the framework of the Innovative Therapies for Children and Adolescents with Cancer Consortium (ITCC), was conducted for patients with refractory/intolerant (R/I) CML or R/R Ph+ ALL after prior imatinib therapy, and identified as the recommended phase 2 dose (RP2D) once daily 60 mg/m^2^ for CML-CP patients and once daily 80 mg/m^2^ for children with accelerated or blastic phase CML (CML-AP/BP) and Ph+ ALL [29]. Based on these results and on the hypothesis that substituting dasatinib for imatinib would lead to more rapid clearance of leukemia and better outcomes for pediatric Ph+ ALL patients, the COG group conducted the ALL0622 phase II trial from 2008 to 2012 on a small cohort of patients (n = 60) (NCT00720109) with the aim being to determine the feasibility and safety profile of adding dasatinib (60 mg/m^2^ continuously from day 15 of induction) to the same chemotherapy backbone previously used in the COG AALL0031 [30]. The results of this trial showed an impressively high rate of end of induction (EOI) complete remission (98%) and end of consolidation (EOC) MRD < 0.01% (89%), which compared favorably with the previous COG study (COG AALL0031) [30]. However, the reported 5-year EFS of 60% was effectively identical to those of both previous imatinib-containing protocols (COG AALL0031 and EsPhALL2004; 5-year EFS 58% and 4-year EFS 61.9%, respectively), not allowing the trial to conclude that dasatinib plus chemotherapy was better than the imatinib plus chemotherapy combination. Notably, this study reinforced the evidence that in the TKI-era the HSCT indication for Ph+ ALL pediatric patients can be limited to high-risk patients. The COG AALL0622 study closed early in order to open the first collaborative Ph+ ALL trial conducted jointly by the COG and EsPhALL trial (NCT01460160, CA180-372/COG AALL1122). This phase II study, the long-term results of which were recently published by Hunger et al. [31], investigated the efficacy of the early and continuous administration of dasatinib in combination with the EsPhALL chemotherapy backbone. The 65.5% 3-year EFS reported was consistent with but not superior to the results of imatinib-containing chemotherapy multi-agent treatment protocols (EsPhALL 2004/2010), despite the very low HSCT rate in CR1 (14%) and the elimination of prophylactic cranial irradiation.

Within the limitation of the small number of patients, the experience of St. Jude Children’s Research Hospital (SJCRH) also clearly showed the advantage of early and continuous exposure to TKS in the treatment of patients with Ph+ ALL. The frontline protocols Total XV (NCT00137111) and Total XVI (NCT00549848) integrated, respectively, imatinib 340 mg/m^2^ and dasatinib 40 mg/m^2^ twice a day from day 22 of induction, in the treatment of newly diagnosed Ph+ ALL patients. In the 11 patients who received TKIs (5 imatinib and 6 dasatinib) the 5-year EFS was 68.6%, comparing favorably with the outcomes of patients treated at SJCRH pre-TKIs (31.6%, *p* = 0.022) [27].

Between 2015 and 2018, the first randomized clinical trial in newly diagnosed pediatric Ph+ ALL patients comparing imatinib 300 mg/m^2^ (n = 97) and dasatinib 80 mg/m^2^ (n = 92), in combination with a modified SJCRH Total XV/XVI chemotherapy backbone, was conducted by the Chinese Children’s Cancer Group (CCCG) (ChiCTR-IPR-14005706). Shen et al. reported, with a median follow-up of 26.4 months, a significantly better outcome for the dasatinib arm; 4-year EFS of 71% vs. 48.9%, and 4-year OS of 88.4% vs. 69.2% were reported for dasatinib and imatinib, respectively [21]. However, there are some caveats to bear in mind when interpreting these results. Firstly, the follow-up of the study was short, considering the slightly high rate of late relapse in the TKI era for Ph+ ALL. Furthermore, the reported results of the imatinib arm were significantly inferior compared to previous COG and EsPhALL study results, which can be only partially explained by the lower imatinib dosing, higher dasatinib dosing and different backbones administered. Considering the results of all these clinical trials, to date there is no conclusive evidence regarding the superiority of dasatinib or imatinib, and both represent, in combination with chemotherapy, the standard of care for treating Ph+ ALL pediatric patients.

Although the prognosis of Ph+ ALL pediatric patients has markedly improved due to the optimization of TKI-containing multi-agent treatment, the outcomes of Ph+ ALL are still inferior to Ph-negative ALL patients and, thus, novel therapeutic improvements are urgently needed. Recently, very favorable results have been obtained in the treatment of adult patients with Ph+ ALL by combining TKIs such as dasatinib and ponatinib with immunotherapeutic approaches such as blinatumomab or inotuzumab [89,90,91,92,93,94,95], paving the way for a possible chemotherapy-free treatment scenario for this specific acute leukemia subtype, including sparing HSCT as a definitive consolidation.

For ABL-class fusion positive ALL, three major frontline pediatric clinical trials including dasatinib have been conducted in recent years. The COG AALL1131 trial (NCT02883049) for patients with high-risk B-ALL was amended in 2016 to add a post-induction dasatinib non-random treatment arm, including post-induction dasatinib (NCT01406756) for those with ABL-class fusion [96]. The preliminary results of this trial were recently reported [32] with a 4-year EFS of 52.5 ± 18% for the HR ABL-class fusion positive patients treated with the incorporation of dasatinib compared to 86.8 ± 0.7% (*p* < 0.0001) of all patients on AALL1131 study. Despite the limitation of a small cohort of patients (n = 22) and the high rate of treatment discontinuation of the prescribed protocol therapy (17/22), these are the first available prospective data from a pediatric trial in ABL-class fusion positive ALL. Additionally, the SJCRH Total therapy XVII study (NCT03117751), started in 2017, explored the effect of adding dasatinib from day 15 of induction to all patients with ABL-class fusion positive ALL identified by RNA-seq, regardless of the patient’s MRD level [97]. Notably, in the total XVII study, T-ALL patients who presented ABL-class fusions with partner genes such as NUP214, ETV6, SLC9A3R1 or MBNL1 were also included in the dasatinib sub-arm jointly with B-ALL ABL-class fusion positive patients. The DFCI ALL 16-001 study (NCT03020030) also evaluated the incorporation of dasatinib in the frontline treatment of very high risk B-ALL with ABL-class fusions other than *BCR::ABL1*. The results of these two former studies have not yet been published.

In pediatrics, the newly opened COG AALL2131/EsPhALL 2022 study (NCT06124157) is exploring an immuno-chemotherapeutic approach with the early introduction of blinatumomab to replace conventional chemotherapy and the combination of fusion-specific TKI therapy for patients with newly diagnosed Ph+ or ABL-class ALL. All patients receive dasatinib with the exception of those with ABL-class ALL harboring PDGFRB treated with imatinib, based upon recent robust data demonstrating differential TKI sensitivity amongst specific ABL-class fusion subgroups [98].

Finally, also the MA-SPORE ALL 2020 study (NCT06336395), ongoing in Malaysa and Singapore, and the NCT06257394 trial, ongoing in South Korea, include dasatinib for the treatment of Ph+ and ABL-class positive ALL pediatric patients.

Although *BCR::ABL1* fusions in patients with T-ALL are rare, high in vitro and ex vivo susceptibility to dasatinib in up to 30% of non-*BCR*::*ABL1*-rearranged T-ALL cases was recently reported [99,100]. Based on this evidence, T-ALL trials including dasatinib have been designed. The phase I/II trial RAVEN (NCT05192889), evaluating the activity of combination chemotherapy with venetoclax and navitoclax in children with R/R ALL, which included dasatinib for children with R/R ABL-class fusions and non-ETP T-ALL, has recently completed its enrollment. Furthermore, the SJALL23T study (NCT06390319) sponsored by the SJCRH, will test whether the addition of dasatinib to the AALL1231 protocol can improve outcomes for children and young adults with newly diagnosed T-ALL without the near-ETP or ETP phenotype.

Notably, a European-based master protocol sponsored by the Princess Maxima Center for Pediatric Oncology (PMC) in collaboration with major European academic consortia (ITCC, iBFM, IntReALL, and EICNHL) has been recently designed (International Proof of Concept Therapeutic Stratification Trial of Molecular Anomalies in Relapsed or Refractory HEMatological Malignancies in Children, HEM-iSMART), with the aim to investigate multiple investigational medicinal products in children, adolescents and young adults (AYAs) with both R/R B- and T-ALL [101]. The HEM-iSMART sub-protocol B phase I/II trial (NCT05751044) will evaluate the safety and efficacy of dasatinib in combination with venetoclax + dexamethasone + cyclophosphamide and cytarabine in children and AYAs with R/R B- and T-ALL, whose leukemia presents with alterations in the MAPK/SRC pathway.

#### 3.1.3. Ponatinib

Ponatinib is a potent third-generation TKI, also active in patients with T315I mutations in the *ABL1* kinase domain, which confer resistance to first- and second-generation TKIs. Currently, ponatinib is approved in adult patients for the second-line treatment of CML and Ph+ ALL and for first-line treatment in the case of a T315I mutation. In the pediatric ALL setting, a phase I/II study exploring the safety and efficacy of ponatinib combined with chemotherapy in pediatric patients with relapsed Ph+ ALL resistant to previous TKI therapies or with the T315I mutation [34], was recently terminated due to dose-limiting toxicities in the phase I (NCT04501614). Ponatinib is currently being tested in an international phase I/II company-sponsored basket trial (INC B 84344) with the aim to assess its safety and efficacy for the treatment of pediatric R/R leukemias (including ALL), lymphomas, and solid tumors (NCT03934372). Additionally, the MD Anderson Cancer Center (MDACC) has designed a phase II adult/pediatric trial, which will include also children aged ≥ 12 years, for testing the combination of ponatinib with mini-hyper CVD chemotherapy and venetoclax in patients with R/R T-ALL (NCT05268003).

#### 3.1.4. Nilotinib

Nilotinib is a second-generation TKI which is currently approved by the FDA and EMA for the treatment of both CML and Ph+ ALL in adults. In children, conversely, its approval is limited to patients with CML-CP either in first-line treatment or R/I to previous TKI treatment. After the phase I study (NCT01077544), which evaluated nilotinib in pediatric R/I CML patients together with R/R Ph+ ALL [33], no subsequent phase II studies in the Ph+ ALL pediatric population have been conducted.

#### 3.1.5. Olverembatinib

Olverembatinib is a novel third-generation TKI which has recently shown promising results in patients with TKI resistant and/or intolerant CML and R/R Ph+ ALL in adults. A pediatric phase I study is currently ongoing in China for children with R/R Ph+ ALL. This phase I study (NCT05495035) is testing the safety and efficacy of olverembatinib in combination with the second-generation BCL2 inhibitor lisaftoclax, and preliminary promising safety and efficacy data have been reported [35].

No clinical trials in children with ALL are currently available for other TKIs. However, pediatric-specific trials of asciminib are being conducted in CML [102] and are planned for Ph+/ABL-class Ph-like ALL.

### 3.2. Janus Kinase Inhibitors

Among Ph-like ALL, the largest class of kinase-activating alterations is characterized by the deregulation/activation of JAK/STAT signaling due to different genetic alterations, including *CRLF2* rearrangements (CRLF2r) with frequent *JAK2* or *JAK1* co-mutation, *JAK2* rearrangements, *EPOR* rearrangements, *SH2B3* deletions, and *IL7R* indels [18]. This pathway represents a major potential therapeutic vulnerability. Based upon robust preclinical data, Janus kinase (JAK) inhibitors, such as ruxolitinib, have been investigated in children and adults with this Ph-like ALL subtype (Table 1).

#### Ruxolitinib

Ruxolitinib is a potent, orally bioavailable inhibitor of the JAK family of kinases with particular efficacy against JAK1 and JAK2. It was first tested as a monotherapy in children with relapsed/refractory solid tumors or hematologic malignancies in the COG ADVL1011 phase 1 study (NCT01164163), which demonstrated a manageable toxicity profile and identified the RP2D as 50 mg/m^2^/dose BID [36]. Ruxolitinib has been subsequently investigated in combination with intensive multi-agent chemotherapy in children and AYAs with newly diagnosed HR CRLF2r/JAK pathway-mutant Ph-like ALL in the COG AALL1521 phase 2 study (NCT02723994). In part 1 of the study, the safety of adding ruxolitinib to post-induction high-risk B-ALL chemotherapy was explored at six different dose levels, and the RP2D of ruxolitinib was identified as 50 mg/m^2^/dose × 14 days on/14 days off per cycle of multi-agent chemotherapy [37]. The part 2/efficacy phase of the study involves evaluating whether the addition of ruxolitinib to the standard of care in high-risk B-ALL chemotherapy backbone may improve outcomes for children and AYAs with Ph-like ALL harboring JAK/STAT pathway lesions, with results anticipated in 2026. Additionally, in the total therapy XVII study (NCT03117751), patients with Ph-like ALL with JAK-STAT signaling activation and with day 15 or day 22 MRD ≥ 5% and all the patients with ETP and MPAL received combination therapy with ruxolitinib. These latter two COG and SJCHR trials completed their enrollment in 2022 and 2023, respectively, and their results are pending. Furthermore, the sub-protocol C of the previously mentioned master protocol HEM-iSMART will evaluate the safety, tolerability, pharmacokinetics (PK), and efficacy of ruxolitinib with venetoclax in combination with dexamethasone, cyclophosphamide, and cytarabine in children and AYAs with R/R ALL with alterations in the IL-7R and/or JAK-STAT signaling pathways [50]. Notably, CRLF2/JAK alterations are also very common in ALL patients with Down syndrome, but these children have been excluded from ruxolitinib-based clinical trials to date.

### 3.3. Proteasome Inhibitors

The ubiquitin–proteasome pathway is essential for the degradation of most proteins and represents, therefore, an actionable pathway, by sensitizing leukemic blasts to apoptosis induced by chemotherapy. Three major proteasome inhibitors, namely bortezomib, carfilzomib, and ixazomib, have been developed and tested in hematological malignant diseases including pediatric ALL (Table 1).

#### 3.3.1. Bortezomib

Bortezomib, as a single agent, was first tested in children with refractory ALL in the phase I study (NCT00077467) sponsored by the COG, which determined the R2PD as 1.3 mg/m^2^/dose, administered twice weekly for 2 weeks followed by a 1-week rest. Little activity as monotherapy was found in this population [38]. Subsequently the Therapeutic Advances in Childhood Leukemia and Lymphoma (TACL) consortium conducted the first phase 1/2 study of bortezomib in combination with chemotherapy (NCT00440726) in R/R pediatric ALL patients [39]. The phase I trial demonstrated that the combination of bortezomib (1.3 mg/m^2^) with a standard 4-drug chemotherapy reinduction backbone (vincristine, dexamethasone, pegylated L-asparaginase, and doxorubicin) (VPLD) was active with acceptable toxicity in pediatric patients with relapsed/refractory ALL. The phase 2 expansion of this combination (n = 22) in highly pretreated relapsed patients (second or more relapse) showed an overall response rate of 73% [40]. Furthermore, the same combination (Bortezomib + VPLD) was tested in the setting of both B- and T-ALL early and very early first relapse in the COG AALL07P1 phase 2 clinical trial (NCT00873093) [41]. Overall CR2 rates were 68 ± 5% for B-ALL patients, with 63 ± 7% for very early relapse (<18 months from diagnosis) and 72 ± 6% for early relapse (18–36 months from diagnosis). Patients with relapsed T-ALL had a particularly encouraging CR2 rate of 68 ± 10%, suggesting the benefit of adding bortezomib in this group of patients [41]. At the same time, a European pediatric phase II trial explored the efficacy of reinduction chemotherapy including bortezomib in a cohort of heavily pretreated patients. Children were randomized 1:1 to bortezomib (1.3 mg/m^2^/dose) administered early or late to a dexamethasone and vincristine backbone. The study reported an overall response rate of 60% with no efficacy differences between the two arms [22]. Based on these results, the I-BFM-SG International Study for Children and Adolescents with Relapsed ALL Consortium (lntReALL) designed the IntReALL 2010 high-risk study (IntReALL HR 2010) which is investigating, in a randomized fashion design, the effect of adding bortezomib to the UK ALLR3 reinduction backbone in high-risk ALL patients in their first relapse (NCT03590171). The results of this large international study, which will provide data on larger cohorts of first-relapsed B- and T-ALL are yet to be published, and interim analysis testing did not reach the superiority or futility criteria. Additionally, the CCCG protocol (CCCG-ALL-2017, NCT04224571) tested the addition of bortezomib during reinduction in combination with chemotherapy in relapsed ALL children (results still unpublished). Concomitantly, two international randomized phase III studies have been carried out, which evaluate the effect of combining bortezomib to multi-agent chemotherapy in the frontline setting of pediatric ALL. The European AIEOP-BFM ALL 2017 study, which has recently completed its accrual, investigated in a randomized fashion whether the addition of bortezomib during the extended consolidation treatment can improve outcomes in early HR B-ALL patients (NCT03643276) based on the stratification of flow of MRD at day 15. The COG group conducted, instead, a phase 3 study with the aim to compare outcomes in children with newly diagnosed T-ALL and T-Lymphoblastic (T-LLy), who were randomly assigned to a modified BFM backbone with/without the bortezomib during induction and delayed intensification (AALL1231, NCT02112916). The results of the AALL1231 trial, recently published by Teachey et al. [42], did not show, overall, a statistically significant improvement of outcomes on the bortezomib arm (4-year EFS: 80.1 ± 2.3% (no bortezomib) versus 83.8 ± 2.1% (bortezomib); *p* = 0.131). Of note, post hoc analyses suggested that bortezomib was effective in T-LLy (4-year EFS: 76.5 ± 5.1% vs. 86.4 ± 4.0%; *p* = 0.041) and not in T-ALL (4-year EFS: 82.9 ± 2.4% vs. 81.5 ± 2.5%; *p* = 0.396), although it is not well understood what the biology behind this might be. In light of these results, multiple subsequent trials incorporated bortezomib as a promising experimental treatment for T-ALL and T-LLy, and results in these larger cohorts must be awaited to better interpret its potential added value. The SJCRH total therapy XVII study (NCT03117751) included bortezomib in the early intensification treatment of patients with no targetable lesions and day 15 or day 22 MRD ≥ 5% or LLy patients without EOI complete response, and the future SJALL23T study will include bortezomib for patients with T-LLy (NCT06390319). The incorporation of bortezomib for newly diagnosed T-LLy is currently also being tested by the ongoing CCCG study (NCT05681260). Interestingly, given preclinical data showing bortezomib and vorinostat (histone deacetylase inhibitor) to be novel active agents in primary KMT2A rearranged (KMT2A-r) infant ALL, the SJCHR also carried out the Total Therapy for Infants with Acute Lymphoblastic Leukemia I study (TINI 1), which incorporated these two agents into an ALL chemotherapy backbone containing dexamethasone, mitoxantrone, and peg-asparaginase during induction and reinduction chemotherapy cycles (NCT02553460). The successor study, TINI 2 (NCT05848687), builds upon the bortezomib/vorinostat backbone by incorporating two cycles of blinatumomab and the menin inhibitor ziftomenib in combination with chemotherapy during reinduction [43].

#### 3.3.2. Carfilzomib

Carfilzomib is a second-generation proteasome inhibitor which demonstrates higher selectivity and potency compared to bortezomib in preclinical ALL models [103]. A global pediatric phase IB/II trial of carfilzomib combined with vincristine, dexamethasone, asparaginase, and daunorubicin (VXLD) as a reinduction therapy for children with R/R ALL has been recently completed (NCT02303821). The dose-escalation phase I study identified 56 mg/m^2^ as the RP2D of carfilzomib and demonstrated the good tolerance of this combination. Encouraging overall response rates (CR/CRp/CRi) of 50% after induction (12/24) and 58% after consolidation (14/24) in the overall cohort of both relapsed B-ALL and first relapsed, nonrefractory T-ALL patients, were reported [44]. The use of the RP2D of carfilzomib with a modified VLXD as a reinduction therapy in pediatric patients with R/R ALL has further been investigated in the phase 2 part of this trial (NCT02303821) [104], for which results are still pending.

#### 3.3.3. Ixazomib

Ixazomib is a third-generation orally bioavailable proteasome inhibitor, with an improved pharmacokinetic and safety profile compared to bortezomib [105]. The TACL consortium has recently carried out a phase 1 trial of oral ixazomib combined with chemotherapy in children and AYAs with relapsed/refractory ALL or lymphoma, and preliminary results were recently reported (NCT03817320) [45]. Ixazomib at the RP2D of 2 mg/m^2^/dose showed an acceptable safety profile and an encouraging early efficacy signal in combination with a VXLD backbone chemotherapy. The phase 2/expansion cohort of the study is ongoing.

### 3.4. BH3 Mimetics

The B-cell lymphoma (BCL-2) family of proteins, including anti-apoptotic (BCL-2, BCL-X_L_, MCL-1) and pro-apoptotic proteins (BAX, BAK and BH3-only), are essential regulators of programmed cell death and the dysregulation of their interplay has been demonstrated to be a key mechanism of leukemic blast survival in both acute myeloid leukemia (AML) and ALL [106]. This evidence has prompted the development, in the last decade, of BH3 mimetic compounds that inhibit the activity of anti-apoptotic BCL-2 member proteins. Two major BCL-2 inhibitors, venetoclax and navitoclax, have emerged in recent years as effective novel therapeutic approaches across several different hematological malignancies including ALL, and new-generation BCL-2 inhibitors molecules are under preclinical and clinical investigation (Table 1).

#### Venetoclax and Navitoclax

Venetoclax is the first-in-class, orally bioavailable, small molecule that potently and selectively inhibits BCL-2 and promotes apoptosis. It is approved for the treatment of different malignant hematological conditions, i.e., newly diagnosed and R/R chronic lymphocytic leukemia (CLL) and AML in combination with hypomethylating agents (HMAs) in adults with newly diagnosed diseases ineligible for intensive chemotherapy. Various preclinical studies have shown the dependence of leukemic lymphoblasts on BCL-2 and BCL-X_L_ and, accordingly, the antileukemic activity of BCL-2/BCL-X_L_ inhibitors in ALL models [107,108,109]. The first-in-child study of venetoclax was a global Phase I study (NCT03236857) evaluating the safety, PK, and preliminary efficacy of venetoclax monotherapy in combination with chemotherapy in pediatric and young adult patients (<25 years) with different R/R hematological and solid malignancies. The results for the ALL cohort were reported by Place et al. [46,110] to be as follows: venetoclax in combination with medium intensity chemotherapy based on the investigators choice for two different backbones (cytarabine-based regimen or vincristine dexamethasone, and PEG-asparaginase (VXL)-based regimen) was well tolerated, with the RP2D identified as an 800 mg adult equivalent dose in combination with chemotherapy, once daily, including a ramp-up of 3-days to mitigate the risk of tumor lysis syndrome, and a promising preliminary efficacy was observed with an ORR of 55% for the VXL-based combination. Concomitantly, the combination of venetoclax with the dual BCL-X_L_/BCL-2 inhibitor navitoclax and chemotherapy was assessed in a phase I dose-escalation study in pediatric and adult patients with R/R ALL or LLy [47] (NCT03181126). This study showed the safety and feasibility of this combination strategy and reported a very promising efficacy with an ORR of 59.6% (n = 28/47) for the entire cohort and 75% (n = 9/12) among pediatric patients. Of note, despite the limitation of the small number of patients, responses were observed across histological and genomic subtypes, including heavily pretreated patients who failed available therapies including HSCT. The pediatric RP2D 25 mg/50 mg navitoclax (depending on weight) with a 400 mg venetoclax adult equivalent dose was identified. The combination of venetoclax and navitoclax has also been investigated by the RAVEN study conducted by SJCRH. In this non-randomized phase I/II clinical trial children with R/R ALL or LLy received venetoclax and navitoclax plus chemotherapy as the first reinduction course (Block 1). Following Block 1, this trial included a phase 1 rolling six design and a phase 2 expansion part to assess the combination dose of venetoclax combinations with either blinatumomab for CD19-postive patients (Block 2B) or navitoclax and high-dose cytarabine for CD19-negative patients (Block 2A) (NCT05192889). Notably, despite its encouraging efficacy, the clinical development of navitoclax for ALL was recently discontinued by the pharmaceutical company.

Furthermore, as previously mentioned, the MDACC trial (NCT05268003) evaluated venetoclax in combination with ponatinib and mini-hyper CVD chemotherapy in R/R T-ALL patients > 12 years of age. The results of these former two studies are still pending. Venetoclax is currently also under investigation in an US multi-institutional phase I trial in both myeloid and lymphoid hematologic malignancies (NCT05292664). The cohort C of this study will include pediatric patients with R/R ALL or LLy and will test venetoclax in combination with chemotherapy; a total of 18 patients are expected to participate in part 1 (dose determination) and an additional 12 will participate in part 2 (dose expansion).

Additional venetoclax-based clinical trials for pediatric ALL are planned to be launched in the near future. The SJCRH will conduct the SJALL23T study (NCT06390319) to evaluate the benefit of adding venetoclax on top of the 4-drug induction of AALL1231 in children with newly diagnosed ETP or near-ETP ALL. The next frontline COG trial AALL2321 (NCT06317662) for infant ALL patients will investigate the efficacy of adding venetoclax on top of the interfant-backbone chemotherapy combined with blinatumomab. Finally, exploring epigenetic approaches in T-ALL, being highly epigenetically driven [111] and by adding synergism of BH3 mimetics in combination with hypomethylating agents, the MDACC plans to carry out a study in R/R pediatric T-ALL patients that evaluates the combination of venetoclax, decitabine, and calaspargase Pegol-mknl (NCT06561074).

### 3.5. MEK Inhibitors

The RAS/RAF/MEK/ERK or MAPK/ERK pathway is a critical signal transduction cascade implicated in the uncontrolled proliferation of many human cancers including ALL. RAS pathway-activating mutations are found in 38% of relapsed pediatric B-ALL patients and additional mutations that activate the RAS signaling cascade (NF1, BRAF, IKZF2, IKZF3, JAK1) have also been found with varying frequencies in relapsed ALL [112]. Preclinical studies showed that RAS pathway mutations confer sensitivity to mitogen-activated extracellular protein kinase (MEK) inhibitors. Two major MEK inhibitors, selumetinib and trametinib, are under clinical investigation for pediatric RAS pathway-mutant ALL (Table 1).

#### 3.5.1. Selumetinib

Based on the preclinical data suggesting the synergistic anticancer effect of the combination of dexamethasone with the MEK 1/2 inhibitor, selumetinib [113], the Cancer Research UK Clinical Trials Unit (University of Birmingham) has sponsored an international, parallel-group, dose-finding with expansion, phase I/II trial (NCT03705507) to assess the selumetinib/dexamethasone combination in adult and pediatric patients with R/R RAS pathway-mutant ALL [114]. The results of this phase I study were recently reported. Although this trial faced various hurdles, including slow recruitment and safety issues concerning severe infections, for which the changing of the dexamethasone dosing and the mandating of antimicrobial prophylaxis were deemed necessary, the preliminary outcome data, with 44% of patients achieving CR (4/9 pts, 3 adults and 1 child), was promising [48].

#### 3.5.2. Trametinib

Trametinib is a reversible and highly selective allosteric MEK1/MEK2 inhibitor, with an indication for the treatment of different solid tumors characterized by MAPK signaling pathway alterations, such as advanced melanoma [115] or low-grade gliomas [116]. Notably, preclinical studies showed that trametinib enhances glucocorticoid responsiveness and is synergistic with the dexamethasone also in T-ALL cell lines [117]. The phase I/II sub-study D of the previously described HEM-iSMART master protocol (NCT05658640), is currently enrolling patients to the test safety and efficacy of trametinib in combination with dexamethasone, cyclophosphamide, and cytarabine in children and AYAs with R/R T-ALL and LBL which harbor alterations in the RAS–RAF–MAPK pathway [101].

### 3.6. Cyclin-Dependent Kinase Inhibitors

The deregulation of cyclin-dependent kinases (CDKs) is associated with the uncontrolled proliferation of cancer cells in a wide spectrum of different malignancies including acute leukemias. CDKs inhibition strategy has therefore emerged as a novel molecularly targeted cancer treatment in recent years. In the setting of pediatric acute leukemias, although there is still limited clinical experience with CDK inhibitors treatment, some clinical data is emerging for both palbociclib and ribociclib (Table 1).

#### 3.6.1. Palbociclib

Palbociclib is an oral CDK4/CDK6 inhibitor which showed promising preclinical efficacy when combined with simultaneous chemotherapy in both B- and T-ALL models [118]. Its safety and preliminary activity in combination with a chemotherapy reinduction backbone was recently investigated by the COG AINV18P1 study (NCT03792256) in both relapsed T-ALL/LLy (1st or greater relapse) and multiply relapsed B-ALL/LLy pediatric patients. Despite the limitation of the small numbers (n = 12), this regimen with palbociclib at the RP2D (50 mg/m^2^/day for 21 days) showed promising efficacy with 42% of ORR in heavily pretreated patients [49]. Another phase I study of palbociclib in combination with chemotherapy was initially started at the SJCRH (RELPALL study, NCT03515200) and is currently sponsored by Standford University (RELPALL2 study, NCT04996160) with the primary objective of confirming the safety of the previously estimated MTD of palbociclib (100 mg/m^2^/day, days 1–5; 11–15; 21–30) in combination with chemotherapy for pediatric patients with R/R ALL.

#### 3.6.2. Ribociclib

Ribociclib is another selective CDK4/CDK6 inhibitor, and its safety and efficacy in children was firstly tested in a phase I study including patients with malignant rhabdoid tumors, neuroblastoma, and other solid tumors [119], and subsequently in the arms A and B of the AcSé-ESMART Trial (NCT02813135) in combination with topotecan-temozolomide or everolimus, respectively, in children with advanced malignancies [50]. In this last study, one patient with a T-ALL with CDKN2A/CDKN2B deletion and PTEN loss was treated with ribociclib and everolimus, achieving a clinical response with a decrease in the number of blasts but ultimately experiencing the progressing disease. The arm M of the AcSé-ESMART trial is currently enrolling patients with malignant hematological conditions harboring alterations in the cell cycle and mTOR pathway evaluating the ribocliclib in combination with everolimus and dexamethasone. Furthermore, ribociclib with this same combination treatment is currently under investigation in children and AYAs with R/R ALL in a phase I study sponsored by the Dana–Farber Cancer Institute (DFCI) (NCT03740334).

### 3.7. mTOR Inhibitors

The phosphatidylinositiol 3-kinase (PI3K), AKT, and mammalian target of rapamycin (mTOR) (PI3K/AKT/mTOR) is a frequently dysregulated signaling pathway in a wide range of hematological conditions including B- and T-cell ALL. The antileukemic activity of rapamycin analogs including everolimus, sirolimus, and temsirolimus have been extensively studied in vitro and in vivo preclinical ALL models providing the rationale for investigating these agents in clinical trials [120,121] (Table 1).

#### 3.7.1. Sirolimus

Sirolimus was first tested as single agent in ≥2nd relapsed pediatric ALL patients in a monocentric phase 1 study (NCT00068302) at the Children’s Hospital of Philadelphia [52], demonstrating no toxic effects, with three out of seven treated patients showing stable disease as best response. Subsequently, two other institutional studies combining sirolimus with glucocorticoids [53] (NCT00874562) and with methotrexate (NCT01162551), respectively, were conducted. Of note, sirolimus in combination with 5 days of prednisone (window therapy prior to starting multi-agent reinduction chemotherapy) showed to suppress MCL-1 expression in vivo, suggesting that mTOR inhibitors may improve sensitivity to glucocorticoids. Next, a single-institution trial sponsored by the Children’s Hospital Medical Center of Cincinnati, combining sirolimus with the UKALLR3 backbone (NCT01658007) was initiated, but terminated due to slow accrual.

#### 3.7.2. Everolimus

Following the promising evidence of adding mTOR inhibitors to chemotherapy, the DFCI sponsored a multi-institutional phase 1b trial (NCT01523977) in first relapse pediatric ALL patients combining everolimus with a standard 4-drug reinduction regimen. The results of this study proved the safety and tolerability of the applied regimen and the high second CR rate of 86% was reported, with 68% of patients achieving MRD < 10^−3^ after reinduction. The RP2D of everolimus in combination with a 4-drug reinduction was established as 5 mg/m^2^/day [54]. Furthermore, a phase I trial (NCT03328104) testing everolimus in combination with a NECTAR regimen (nelarabine, cyclophosphamide, and etoposide) in relapsed T-ALL/LLy was recently completed, and the results are pending. Finally, everolimus is also under investigation in combination with ribociclib and dexamethasone in a phase I study sponsored by the DFCI (NCT03740334).

#### 3.7.3. Temsirolimus

Concomitantly to the everolimus DFCI study, the COG ADVL1114 phase I study (NCT01403415) explored the safety and preliminary activity of the mTOR inhibitor temsirolimus (three weekly doses) in combination with an intensive reinduction chemotherapy (UK ALLR3) for 2nd or greater relapse of pediatric ALL. Although 7 of the 15 evaluable patients had a CR or CRi, this treatment combination resulted in excessive toxicity at all dose levels of temsirolimus tested and it was then considered not safe in children with relapsed ALL [55]. The high toxicity shown by the COG ADVL1114 study with 4-drug reinduction therapy prompted the TACL consortium to design a subsequent trial (NCT01523977) of temsirolimus (two weekly doses) in combination with an alternative cyclophosphamide- and etoposide-based chemotherapy backbone in the same patient population. This combination showed an acceptable safety profile and an ORR of 47%. The RP2D of temsirolimus with this chemotherapy was identified at 15 mg/m^2^ [56].

### 3.8. Epigenetic Modifying Agents

Epigenetic alterations involving histone modification or DNA methylation are quite frequent in ALL and have been associated with treatment resistance and relapse risk, leading to the development of therapeutic strategies targeting these epigenetic aberrations. Globally, these agents are referred to as “epigenetic modifying agents” and include histone deacetylase inhibitors (HDACi) and DNA methyltransferase inhibitors (DNMTi) or HMAs (Table 1).

### 3.9. Histone Deacetylase Inhibitors (HDACi)

HDAC inhibitors can be classified into structurally diverse classes, including hydroxamic acids, such as vorinostat and panobinostat, cyclic peptides (e.g., romidepsin) and benzamides (i.e., entinostat). Among these drugs, vorinostat is the one in which clinical development in the context of pediatric ALL is more advanced. The combination of HDACi and DNMTi was shown synergistic in in vitro ALL models [122]; hence, these agents have more frequently been investigated in combination than as single agents.

#### Vorinostat

Vorinostat was first tested in children with R/R malignancies, including ALL, by the phase I COG study (NCT00217412), which identified the RP2D as 230 mg/m^2^/d in monotherapy [57]. Next, a monocentric pilot phase II study (NCT00882206), sponsored by the University of Minnesota, investigated the tolerability and efficacy of the combination vorinostat plus decitabine followed by a standard reinduction chemotherapy in pediatric and adults R/R ALL patients [58]. This regimen was tolerable and demonstrated a quite encouraging clinical benefit in a heavily pretreated cohort of patients with 5 out of 13 patients achieving CR and MRD negativity. Based on these results, a subsequent pediatric trial was carried out (NCT01483690) by the TACL consortium to evaluate the effect of an extended schedule of decitabine and vorinostat prior to and concomitantly with an intensive chemotherapy backbone in pediatric and AYA R/R ALL patients. Unfortunately, despite encouraging response rates and pharmacodynamics, this regimen was determined to be not feasible in B-ALL due to the high incidence of significantly infectious toxicities [59]. Furthermore, vorinostat is under clinical evaluation in combination with bortezomib in infants with KMT2A-r ALL, as previously discussed in Section 3.3.1.

### 3.10. Hypomethylating Agents (HMAs)

DNA methyltransferase inhibitors, azacitidine and decitabine, have been widely investigated in recent years in hematological cancers, mainly in the setting of myeloid malignancies but increasingly also in specific ALL groups such as KMT2A-r ALL and T-ALL, for which there exists a strong biological rationale and preclinical evidence for the use of a hypomethylating treatment strategy [106,123,124].

#### 3.10.1. Azacitidine

The first phase I clinical trial (NCT01861002) testing azacitidine combined with chemotherapy in children with relapsed/refractory acute leukemias (mainly AML) was conducted by the TACL consortium and demonstrated the tolerability of 5 days of azacitidine (75 mg/m^2^ per day, subcutaneously) followed by chemotherapy [60]. Some years later, the COG conducted the AALL15P1 single-arm groupwide pilot trial (NCT02828358), testing the hypothesis that the addition of 5 days of azacitidine treatment immediately prior to the start of chemotherapy, in 4 post-induction courses, could enhance chemosensitivity of infants with newly diagnosed KMT2A-r ALL. This trial showed that the addition of azacitidine was well-tolerated and led to decreased DNA methylation, but resulted in similar outcomes to similar infants treated with chemotherapy in the prior COG AALL0631 trial (3-year EFS 34.7%) [61].

#### 3.10.2. Decitabine

Decitabine was initially investigated by the COG consortium in a phase I trial in children with relapsed/refractory AML or ALL, which was terminated due to slow accrual (NCT00042796, unpublished). Another phase 1/2 study (NCT00349596) conducted in both children and adults with R/R ALL demonstrated the safety and encouraging clinical activity of decitabine alone and in combination with hyper-CVAD chemotherapy [62]. Subsequently, decitabine has been investigated in combination with vorinostat as an epigenetic modifying strategy plus chemotherapy (NCT00882206, NCT01483690), as previously discussed in Section Vorinostat. Finally, decitabine will be studied in combination with venetoclax and calaspargase pegol-mknl in children with relapsed/refractory T-ALL or LLy via a single-institution trial at MDACC (NCT06561074).

### 3.11. Molecularly Targeted Treatment in Pediatric KMT2A-r ALL

*KMT2A* is a central regulator of target genes that drive leukemogenic transformation. KMT2A-r ALL represents one of the more aggressive and unfavorable molecular subtypes of ALL. It typically occurs in infant patients and is associated with dismal outcomes in current chemotherapy-based treatment strategies [125,126].

Different precision medicine approaches have, therefore, been explored to address this large unmet clinical need, including the inhibition of signaling pathways (i.e., FLT3 inhibition) and the modulation of aberrant epigenetic programs (i.e., hypomethylating treatment [61] and DOT1L inhibition [127]); however, these efforts have provided only very limited benefits for these patients. Novel treatment strategies, such as immunotherapy (e.g., blinatumomab [128]), BH3 mimetics agents, and menin inhibitors (as well as their combinations) have recently emerged as promising therapeutic approaches for this very difficult-to-treat ALL subtype (Table 1).

### 3.12. FLT3 Inhibitors

The use of *FLT3* inhibitors (FLT3i) for the treatment of acute leukemias mainly concerns AML, which represents nowadays a treatment paradigm for *FLT3*-mutated patients [68,129]. Nevertheless, the evidence of the striking overexpression of *FLT3* in KMT2A-r infant ALL and the preclinical data of *FLT3* inhibition strategy efficacy in KMT2A-r ALL cells [130] has prompted clinical investigation of FLT3i also in KMT2A-r ALL.

#### 3.12.1. Midostaurin

The first-in-child phase I study (NCT00866281) of midostaurin was conducted in both R/R AML and KMT2A-r ALL patients, establishing the recommended dose for expansion as 30 mg/m^2^ twice daily [63]. Nevertheless, in KMT2A-r patients (n = 14) no clinical efficacy was observed and no further studies with midostaurin have thereby been conducted in ALL.

#### 3.12.2. Lestaurtinib

In 2008, the COG started a phase III study to test the hypothesis that the addition of the FLT3i lestaurtinib to post-induction chemotherapy would improve EFS (AALL0631, NCT00557193) in KMT2Ar ALL infant patients, for which the results were recently published [64]. Though, overall, this randomized study did not show any improvement outcomes in KMT2Ar infants treated with chemotherapy plus lestaurtinib compared to chemotherapy only (3-year EFS 36 ± 6% vs. 39 ± 7%, *p* = 0.67), it demonstrated that in the subgroup of patients showing potent pharmacodynamic FLT3-inhibition and higher ex vivo sensitivity to FLT3i, the addition of lestaurtinib was beneficial.

### 3.13. Menin Inhibitors

Recently, the major translational effort for precision medicine in KMT2A-r leukemias has been focusing on a novel molecularly targeted class of small molecules, namely the menin inhibitors. These are oral and selective agents, able to disrupt the interaction between the chromatin adapter menin, and the KMT2A complex. Two different agents, revumenib and ziftomenib, are currently under early clinical-stage investigation in adults and children with both AML and ALL harboring KMT2A rearrangements [131,132] (Table 1).

#### 3.13.1. Revumenib

The AUGMENT-101 study (NCT04065399), opened in 2019, was the first clinical trial testing menin inhibitor revumenib single agents in acute leukemias, and led to the FDA approval of the drug in 2024 for relapsed/refractory acute leukemia with KMT2A rearrangement in patients older than 1 year. This trial included both adult and pediatric patients with R/R KMT2A-r or NPM1-mutated (NPM1m) patients, and the results of the phase I part were recently published [65,133]. In total, 80 patients, including 18 pediatric patients, with R/R KMT2A-r acute leukemias (AML and ALL) were treated across six dose-escalation cohorts, and a RP2D of 276 mg twice a day, without a strong CYP3A4 inhibitor, was established (for patients treated with strong CYP3A4 inhibitors, i.e., azoles, the RP2D was 163 mg twice a day). Preliminary results of the pivotal phase 2 study were also reported. The interim analysis for the efficacy population (n = 57) of pooled adult and pediatric patients with *KMT2A*-r acute leukemia showed a combined CR + CRh of 22.8% and ORR of 63.2%, consistent in both adult and pediatric patients, exceeding the protocol-defined null hypothesis of 10% (primary endpoint); therefore, the *KMT2A*-r cohorts were stopped early for efficacy [66,134,135]. Based on these promising results, the AUGMENT-102 (NCT05326516) study was launched and has recently completed its recruitment. This phase I trial investigated the safety of revumenib in combination with chemotherapy (FLA regimen) in both adults and children with *KMT2A*-r, *NUP98*-r, or *NPM1*-m acute leukemia. Preliminary results of this mostly pediatric population (n = 27, median age 6 years; range 0.8–78) were recently reported, showing preliminary efficacy results similar to that observed with revumenib monotherapy [67]. The publication of the most consolidated results of this trial is expected soon, and together with other ongoing studies—mostly conducted in the context of R/R AML in adult patients (NCT06226571) [136]—will help to highlight the actual benefit of menin inhibitors therapy in combination with intensive chemotherapy for KMT2Ar, NUP98r, and NPM1m leukemias. Currently, the COG consortium is running a phase II study (AALL2121, NCT05761171) to test the safety and efficacy of revumenib in combination with chemotherapy in treating infants and children (aged > 1 month to <6 years) with *KMT2A*-r relapsed/refractory ALL, which estimates the enrollment of up to 78 patients. Furthermore, a phase I study sponsored by the City of Hope Medical Center is investigating the safety and tolerability of revumenib as a maintenance therapy in patients (>2 years old) with *KMT2A*-r or NPM1-m acute leukemia after HSCT (NCT06575296).

#### 3.13.2. Ziftomenib

Ziftomenib is currently under clinical investigation in the adult population in the KOMET-001 study. This first-in-human study tested ziftomenib in patients with relapsed/refractory AML and demonstrated a manageable safety profile and preliminary clinical activity in heavily pretreated patients with *KMT2A*-r or *NPM1*-m AML, warranting further investigation of this agent as a monotherapy and in combination with rational therapeutic partners [137,138,139]. Notably, the CR rate differed between subgroups, with NPM1-mutant patients showing substantially higher response rates to ziftomenib compared to those with KMT2A rearrangements. In the pediatric setting three different ongoing or planned studies aim to evaluate ziftomenib in patients with *KMT2A*-r ALL. The TINI 2 study (NCT05848687), sponsored by the Stanford University, includes ziftomenib as part of a multi-agent therapy including chemotherapy, bortezomib, vorinostat, and blinatumomab [43] for the treatment of infant patients. The ITCC-101/APAL2020K (NCT06376162) is a phase 1 trial designed to assess the safety and determine the RP2D of ziftomenib in combination with chemotherapy for children with relapsed/refractory KMT2A-r, NUP98-r, or NPM1-m acute leukemias [68], in the context of a pediatric obligation (Pediatric investigation plan, EMA; Pediatric study plan, FDA). Finally, the MDACC is enrolling patients from 2 to 21 years-old in a phase I study investigating the combination of ziftomenib, venetoclax, and azacitidine in relapsed/refractory KMT2A-r ALL (NCT06397027).

Other menin inhibitors are being tested in adult patients with potential pediatric-specific investigations planned in the future. Bleximenib, for instance, is currently being tested in a pediatric cohort (12 years and older) of the phase 1/2 study (cAMeLot-1) for acute leukemia patients harboring *KMT2A, NPM1, NUP98*, or *NUP214* alterations (NCT04811560).

## 4. Conclusions and Future Directions

**Figure 3:**
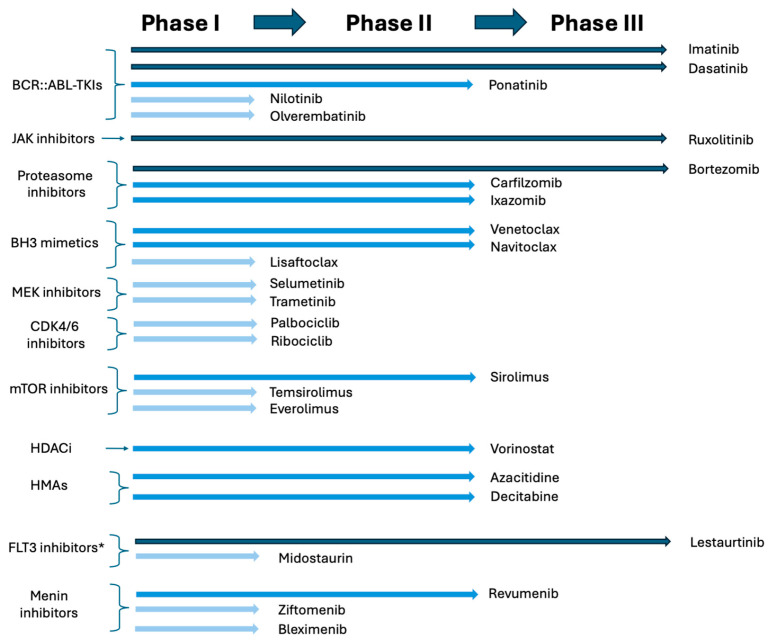
Current stage of clinical drug development in molecularly targeted agents in pediatric ALL. * Though FLT3 inhibitors have been tested in ALL pediatric clinical trials in the past, these agents are not routinely added to chemotherapy for pediatric ALL patients at this time.

In summary, the clinical development of molecularly targeted therapies for pediatric ALL has achieved notable successes, as well as several setbacks. Its landscape remains rapidly evolving, simultaneously making exciting progress and posing new challenges (Figure 3).

There persists an urgent need to develop more effective frontline and salvage treatments for patients with high-risk genetic subtypes of ALL and/or relapse, and to replace toxic therapy elements with less toxic ones yet retain efficacy. In this context, the integration of molecularly targeted agents combined with immunotherapies may represent the next therapeutic paradigm, as exemplified by the recent outstanding success of adult Ph+ ALL treatment combining dasatinib or ponatinib with blinatumomab [140]. Among the emerging rational combinations of molecularly targeted therapy and immunotherapy in the context of ALL, the integration of a menin inhibitor with blinatumomab in KMT2Ar patients appears particularly intriguing. Furthermore, the combination of venetoclax and inotuzumab showed promising activity in preclinical models [141] and in clinical results in adults with R/R ALL [142]. Importantly, future clinical trials should integrate immune monitoring strategies to assess the immunological impact of small molecules. In this context, careful consideration will be required regarding the potential overlapping toxicities between these two therapeutic approaches.

Equally critical is the continued elucidation of relevant biological biomarkers and in-depth genetic diagnostics refinement to enable patient stratification and guide treatment decisions.

There is a growing recognition of the need to introduce molecularly targeted agents earlier in the frontline setting for high-risk genetic patients rather than restricting their use to relapsed disease, as preventing relapse remains the ultimate therapeutic goal rather than treating it. Accordingly, there is the need to accelerate regulatory pathways to enable more rapid approvals in newly diagnosed pediatric patients, ensuring timely access to potentially transformative treatments. An effective strategy to expedite pediatric drug development could involve lowering the minimum eligible age earlier in phase 1 studies, once a recommended phase 2 dose has been defined in adults. This strategy, as exemplified in the revumenib development program, is particularly justified when the underlying genetic abnormalities are shared across pediatric and adult populations. Subsequently, it needs to be discussed whether a program in relapse is needed, or what the minimum dataset is to move the drug into frontline treatment. This may also imply that Pediatric Study Plans with FDA and EMA should contain placeholders for pivotal trials in newly diagnosed children. Drug development in pediatric oncology continues to face significant hurdles, including limited commercial incentives and smaller patient populations compared to adults. Initiatives, such as ACCELERATE, aiming to strengthen the collaboration and active dialogue amongst pediatric oncologists, pharmaceutical partners, regulators, and patient advocates [143], play a key role in addressing these barriers by fostering collaborative and efficient pediatric trial platforms. In conclusion, it is imperative to accelerate the movement of transformative agents to frontline settings, particularly those with game-changing potential such as the *BCR::ABL1*-directed TKIs and menin inhibitors reviewed in this review, to improve cure rates and reduce short- and long-term toxicities for children with ALL.

## Figures and Tables

**Table 1:** Clinical trials including small molecule inhibitor therapies in pediatric ALL.

Trial ID	Study Name	Status	Phase	Disease	Enrollment Period	N. Patients */ Age Eligibility (Years)	Key Results	Reference
***BCR*** ***::ABL1*** **-directed TKIs**								
**Imatinib**								
NCT00004932	-	Completed	1	CML, Ph+ ALL	2000–2001	31/up to 21	Good tolerance at doses ranging from 260 to 570 mg/m^2^	Champagne et al. [23]
NCT00022737	COG AALL0031	Completed	3	Ph+ ALL	2002–2006	91/1–21	5-year EFS 71% (Cohort 5)	Schultz et al. [24]
NCT00287105	EsPhALL 2004	Completed	2	Ph+ ALL	2004–2009	160/1–17	4-year EFS 72.9% (Imatinib) vs. 61.7% (no imatinib) (*p* 0.24) in good-risk patients	Biondi et al. [25]
NCT00287105	EsPhALL 2010	Completed	2	Ph+ ALL	2010–2014	155/1–17	5-year EFS 62.7% in good-risk patients and 46.3% in poor-risk patients	Biondi et al. [26]
NCT00137111	Total Therapy XV	Completed	3	Ph+ ALL	2004–2007	5/1–18	5-year EFS 68.6% (Imatinib + Dasatinib patients)	Jeha et al. [27]
NCT03007147	EsPhALL 2017/ COG AALL1731	Active, not recruiting	3	Ph+ ALL, ABL-class ALL	2017–2024	475/1–17	-	-
NCT04307576	ALLTogether1	Recruiting	3	ABL-class ALL	2019-present	6430/0–45	-	-
**Dasatinib**								
NCT00316953	CA180-038/ COG-ADVL0516	Completed	1	CML, Ph+ ALL, solid tumor	2006–2009	39/1–21	MTD 85 mg/m^2^ (twice daily)	Aplenc et al. [28]
NCT00306202	CA180-018/ ITCC-005	Completed	1	R/I CML, R/R Ph+ ALL	2006–2009	58/1–21	RP2D 80 mg/m^2^ (once daily)	Zwaan et al. [29]
NCT00720109	COG AALL0062	Completed	2/3	Ph+ ALL	2008–2012	60/1–30	5-year EFS 60% in good-risk patients and 67% in high-risk patients	Slayton et al. [30]
NCT00549848	Total therapy XVI	Completed	3	Ph+ ALL	2007–2012	6/1–18	5-year EFS 68.6%	Jeha et al. [27]
NCT01460160	CA180-372/ COG AALL1122	Completed	2	Ph+ ALL	2012–2014	106/1–17	3-year EFS 65.5%	Hunger et al. [31]
ChiCTR-IPR-14005706	-	Completed	3	Ph+ ALL	2015–2018	97 (ima) + 92 (dasa)/0–18	4-year EFS 49% (imatinib) vs. 71% (dasatinib) (*p* 0.005)	Shen et al. [21]
NCT03117751	Total therapy XVII	Active, not recruiting	2/3	ABL-class ALL (B- and T-ALL)	2017–2023	790/1–18	-	-
NCT01406756	COG AALL1131	Active, not recruiting	3	ABL-class ALL	2016–2019	22/1–31	4-year EFS 52.5%	Salzer et al. [32]
NCT03020030	DFCI 16-001	Active, not recruiting	3	ABL-class ALL	2017–2022	560/1–21	-	-
NCT06336395	MA-Spore 2020	Recruiting	3	Ph+ ALL, ABL-class ALL	2020–present	500/up to 40	-	-
NCT06257394	-	Recruiting	2	Ph+ ALL	2024–present	74/1–19	-	-
NCT05192889	RAVEN	Active, not recruiting	1/2	R/R ABL-class ALL, R/R non-ETP ALL	2022–2024	35/4–30	-	-
NCT06124157	EsPhALL 2022/ COG AALL2131	Not yet recruiting	3	Ph+ ALL, ABL-class ALL	-	222/1–46	-	-
NCT06390319	SJALL23T	Not yet recruiting	2	Non-ETP T-ALL	-	100/1–18	-	-
NCT05751044	HEM-iSMART-B	Not yet recruiting	1/2	T-ALL R/R	-	26/1–21	-	-
**Nilotinib**								
NCT01077544	-	Completed	1	R/I CML, Ph+ ALL	2011–2015	15/1–18	RP2D 230 mg/m^2^ (twice daily)	Hijiya et al. [33]
**Ponatinib**								
NCT04501614	-	Terminated	1|2	R/R Ph+ ALL, ABL-class ALL	2020–2024	11/1–21	-	Matloub et al. [34]
NCT03934372	-	Recruiting	1|2	R/R leukemia, lymphomas, solid tumors	2020-present	60/1–17	-	-
NCT05268003	MDACC 2021-0802	Active, not recruiting	2	R/R T-ALL	2020-present	26/12 and older	-	-
**Olverembatinib**								
NCT05495035	-	Active, not recruiting	1	Ph+ ALL	2022–2024	10/0–18	-	Zhang et al. [35]
**JAK inhibitors**								
**Ruxolitinib**								
NCT01164163	ADVL1011	Completed	1	R/R leukemia and solid tumors	2010–2012	49/1–18	RP2D 50 mg/m^2^ (BID)	Loh et al. [36]
NCT02723994	AALL1521 (INCB18424-269)	Active, not recruiting	2	HR Ph-like B-ALL	2016–2022	171/1–21	-	Tasian et al. [37]
NCT03117751	Total therapy XVII	Active, not recruiting	2/3	HR Ph-like B-ALL (B- and T-ALL)	2017–2023	790/1–18	-	-
NCT05745714	HEM-iSMART-C	Not yet recruiting	1/2	R/R T-ALL	-	26/1–21	-	-
**Proteasome inhibitors**								
**Bortezomib**								
NCT00077467	COG-ADVL0317	Completed	1	R/R ALL	2004–2005	12/1–18	RP2D 1.3 mg/m^2^/dose (twice weekly for 2 weeks followed by a 1-week rest)	Horton et al. [38]
NCT00440726	T2005-003	Completed	1/2	R/R ALL	2006–2011	22/1–22	ORR 73%	Messinger et al. [39,40]
NCT00873093	COG AALL07P1	Completed	2	1st R ALL	2009–2013	135/1–18	ORR 68 ± 5% B-ALL; 68 ± 10% T-ALL	Horton et al. [41]
2009-014037-25 (EudraCTnumber)	ITCC 021/I-BFM-SG	Completed	2	R/R ALL	2010–2014	29/0.5–19	ORR 60%	Kaspers et al. [22]
NCT03590171	IntReALL HR 2010	Recruiting	2	1st R ALL	2017–present	250/0–17	-	-
NCT02112916	COG AALL1231	Active, not recruiting	3	T-ALL and T-LLy (1st line)	2014–2017	824/1–30	4-year EFS 80.1% (no bortezomib) vs. 83.8% (bortezomib) (*p* 0.131)	Teachey et al. [42]
NCT04224571	CCCG-ALL-2017 relapse	Completed	2	R/R ALL	2018–2023	208/3 months-21 yrs	-	-
NCT03643276	AIEOP-BFM ALL 2017	Active, not recruiting	3	B- and T-ALL (1st line)	2018–2024	5000/0–17	-	-
NCT03117751	Total therapy XVII	Active, not recruiting	2/3	B- and T-ALL (1st line)	2017–2023	790/1–18	-	-
NCT06390319	SJALL23T	Not yet recruiting	2	Non-ETP T-ALL	-	100/1–18	-	-
NCT05681260	CCCG-T-LBL-2023	Recruiting	3	T-LLy	2023–present	200/12–18	-	-
NCT02553460	TINI I	Active, not recruiting	1/2	Infant ALL	2017–2021	50/up to 1 year	3-year EFS 56.5%	Gruber et al. [43]
NCT05848687	TINI II	Not yet recruiting	1/2	Infant ALL	-	90/up to 1 year	-	-
**Carfilzomib**								
NCT02303821	CFZ008	Completed	1/2	R/R ALL	2015–2024	141/1–21	ORR 50%	Burke et al. [44]
**Ixazomib**								
NCT03817320	T2017-002	Active, not recruiting	1/2	R/R ALL	2019–2024	24/1–21	RP2D 2 mg/m^2^/dose	Schafer et al. [45]
**BH3 mimetics**								
**Venetoclax**								
NCT03236857	M13–833	Completed	1/2	R/R ALL	2017–2023	31/0–25	ORR 55%	Place et al. [46]
NCT03181126	-	Completed	1/2	R/R ALL and LLy	2017–2019	47/4 yrs and older	ORR 60%	Pullarkat et al. [47]
NCT05192889	RAVEN	Active, not recruiting	1/2	R/R ALL	2022–2024	35/4–30	-	-
NCT05268003	MDACC 2021-0802	Active, not recruiting	2	R/R T-ALL	2022–present	26/12 yrs and older	-	-
NCT05292664	-	Active, not recruiting	1	R/R ALL	2023–present	13/1–40	-	-
NCT06317662	AALL2321	Not yet recruiting	2	Front line Infant ALL	-	153/up to 1 year	-	-
NCT06390319	SJALL23T	Not yet recruiting	2	Front line ETP/near ETP ALL	-	100/1–18	-	-
NCT05751044	HEM-iSMART-B	Not yet recruiting	1/2	R/R T-ALL	-	26/1–21	-	-
NCT05745714	HEM-iSMART-C	Not yet recruiting	1/2	R/R T-ALL	-	26/1–21	-	-
NCT06561074	MDACC 2022-0416	Not yet recruiting	2	R/R T-ALL	-	22/1–21	-	-
**Navitoclax**								
NCT03181126	-	Completed	1/2	R/R ALL and LLy	2017–2019	47/4 yrs and older	ORR 60%	Pullarkat et al. [47]
NCT05192889	RAVEN	Active, not recruiting	1/2	R/R ALL	2022–2024	35/4–30	-	-
**Lisaftoclax**								
NCT05495035	-	Active, not recruiting	1	Ph+ ALL	2022–2024	10/0–18	-	Zhang et al. [35]
**MEK inhibitors**								
**Selumetinib**								
NCT03705507	SeluDex, ITCC-063	Terminated	1	R/R ALL	2018–2023	12/All ages	CR 44%	Vormoor et al. [48]
**Trametinib**								
NCT05658640	HEM-iSMART D	Recruiting	1/2	R/R T-ALL	2024–present	26/1–21	-	-
**CDK4/6 inhibitors**								
**Palbociclib**								
NCT03792256	AINV18P1	Completed	1	R/R ALL	2019–2021	12/1–31	50 mg/m^2^/day for 21 days; ORR 42%	Raetz et al. [49]
NCT03515200	RELPALL	Terminated	1	R/R ALL	2018–2020	12/up to 21	-	-
NCT04996160	RELPALL2	Recruiting	1	R/R ALL	2021–present	22/up to 25	-	-
**Ribociclib**								
NCT02813135	AcSé-ESMART sub-trial B	Completed	1/2	R/R leukemia	2016–2019	31/up to 18	-	Bautista et al. [50]
NCT02813135	AcSé-ESMART sub-trial M	Recruiting	1/2	R/R leukemia	2021–present	35/up to 18	-	Geoerger et al. [51]
NCT03740334	18-328	Active, not recruiting	1	R/R ALL	2019–2023	45/1–30	-	-
**mTOR inhibitors**								
**Sirolimus**								
NCT00068302	-	Terminated	1	R/R ALL	2003–2009	10/Up to 21	-	Rheingold et al. [52]
NCT00874562	-	Completed	1	R/R ALL	2007–2009	6/1 year and older	-	Schlis et al. [53]
NCT01162551	-	Completed	2	R/R ALL	2010–2017	5/up to 25	-	-
NCT01658007	-	Terminated	1	R/R ALL	2012–2017	3/up to 30	-	-
**Everolimus**								
NCT01523977	-	Completed	1	R/R ALL	2012–2017	22/18 months-21 years	RP2D 5 mg/m^2^/day; CR 86%	Place et al. [54]
NCT03328104	Aflac LL1602 ENCERT	Completed	1	R/R T-ALL	2018–2023	8/2–29	-	-
NCT03740334	18-328	Active, not recruiting	1	R/R ALL	2019–2023	45/1–30	-	-
**Temsirolimus**								
NCT01403415	ADVL1114	Completed	1	R/R ALL	2012–2014	13/1–21	-	Rheingold et al. [55]
NCT01614197	TACL 2014-001	Completed	1	R/R ALL	2015–2019	16/1–21	RP2D 15 mg/m^2^; ORR of 47%	Tasian et al. [56]
**HDAC inhibitors**								
**Vorinostat**								
NCT00217412	-	Completed	1	Solid/hematologic cancers	2005–2009	64/1–21	RP2D 230 mg/m2/d	Fouladi et al. [57]
NCT00882206	-	Terminated	2	R/R ALL	2009–2013	13/2–60	ORR 46.2%	Burke et al. [58]
NCT01483690	TACL T2009-003	Terminated	1/2	R/R ALL	2011–2015	23/1–21	-	Burke et al. [59]
NCT02553460	TIN I	Active, not recruiting	1/2	Infant ALL	2017–2021	50/up to 1 year	3-year EFS 56.5%	Gruber et al. [43]
NCT05848687	TINI II	Not yet recruiting	1/2	Infant ALL	-	90/up to 1 year	-	-
NCT03117751	Total therapy XVII	Active, not recruiting	2/3	B- and T-ALL (1st line)	2017–2023	790/1–18	-	-
**DNA methyltransferase inhibitors**								
**Azacitidine**								
NCT01861002	TACL T2011-002	Completed	1	R/R ALL	2013–2014	15/1–21	RP2D 75 mg/m^2^/day	Sun et al. [60]
NCT02828358	COG AALL15P1	Completed	2	KMT2A-r infant R/R ALL	2017–2022	78/up to 1 year	3-year EFS 34.7%	Guest et al. [61]
NCT06397027	-	Recruiting	1	R/R AML and ALL (KMT2A-r, NPM1-m or NUP98-r)	2024–present	22/2–21	-	-
**Decitabine**								
NCT00042796	-	Terminated	1	R/R leukemia	2002–2005	21/up to 21	-	-
NCT00349596	-	Completed	1/2	R/R ALL	2006–2014	40/All ages	-	Benton at al. [62]
NCT00882206	-	Terminated	2	R/R ALL	2009–2013	13/2–60	ORR 46.2%	Burke et al. [58]
NCT01483690	TACL T2009-003	Terminated	1/2	R/R ALL	2011–2015	23/1–21	-	Burke et al. [59]
NCT06561074	MDACC 2022-0416	Not yet recruiting	2	R/R T-ALL	-	22/1–21	-	-
**FLT3 inhibitors**								
**Midostaurin**								
NCT00866281	CPKC412A2114	Terminated	1/2	R/R AML and ALL	2009–2014	22/2–18	RDE 60 mg/m^2^ BID	Zwaan et al. [63]
**Lestaurtinib**								
NCT00557193	COG AALL0631	Completed	3	KMT2A-r ALL	2008–2014	210/up to 1 year	3-year EFS 36 ± 6% (chemo + lestaurtinib) vs. 39 ± 7%, (chemo-only) (*p* 0.67)	Brown et al. [64]
**Menin inhibitors**								
**Revumenib**								
NCT04065399	AUGMENT-101	Recruiting	1/2	R/R AML and ALL (KMT2A-r, NPM1 or NUP98-r)	2019-present	94/30 days and older	CR + CRh 22.8% and ORR 63.2% (57 patients)	Issa et al. [65,66]
NCT05326516	AUGMENT-102	Completed	1	R/R AML and ALL (KMT2A-r, NPM1-m or NUP98-r)	2022–2024	30/30 days and older	-	Shukla et al. [67]
NCT05761171	COG AALL2121	Active, not recruiting	2	R/R KMT2A-r ALL	2024–present	78/1 month- 6 years	-	-
NCT06575296	-	Recruiting	1	KMT2A-r and NPM1-m ALL	2025-present	27/2 years and older	-	-
**Ziftomenib**								
NCT06397027	-	Recruiting	1	R/R AML and ALL (KMT2A-r, NPM1-m or NUP98-r)	2024–present	22/2–21	-	-
NCT06376162	ITCC-101/APAL2020K	Recruiting	1	R/R AML and ALL (KMT2A-r, NPM1-m or NUP98-r)	2024–present	20/0–21	-	Salzer et al. [68]
NCT05848687	TINI II	Not yet recruiting	1/2	Infant ALL	-	90/up to 1 year	-	
**Bleximenib**								
NCT04811560	cAMeLot-1	Recruiting	1/2	R/R AML and ALL (KMT2A-r, NPM1-m or NUP98-r)	2024–present	400/12 years and older	-	-

* If the trial is still recruiting or the results are not yet available, enrolment is estimated (source clinicaltrial.gov). Abbreviations: ALL: acute lymphoblastic leukemia; CML: chronic myeloid leukemia; CR: complete remission; CRh: complete remission with partial hematologic recovery; EFS: event-free survival; ETP: early T-progenitor; MTD: maximum tolerated dose; ORR: overall response rate; Ph+: Philadelphia chromosome-positive; RDE: recommended dose escalation; RP2D: recommended phase 2 dose; R/R: relapsed/refractory.

**Table 2:** Toxicity profiles of small molecule inhibitor therapies organized by drug class.

	Main Toxicities	References
*BCR::ABL1*-direcetd tyrosine kinase inhibitors	Hematologic: cytopenia. Gastrointestinal: nausea, vomiting, diarrhea, abdominal pain. Musculoskeletal: muscle cramps, musculoskeletal pain, myalgia. Dermatologic: skin rash, pruritus, depigmentation, photosensitivity. Constitutional: fatigue, headache. Fluid retention: edema, pleural or pericardial effusion (particularly associated with dasatinib). Cardiovascular: arterial/venous thrombosis (specifically for ponatinib). Endocrinologic: thyroid dysfunction, metabolic bone disorders, growth retardation.	[23,29,69,70,71,72]
JAK inhibitors	Hematologic: cytopenia. Increased infection risk. Gastrointestinal: diarrhea, nausea. Constitutional: fatigue, headache. Skin: rash	[36,73]
Proteasome inhibitors	Hematologic: cytopenia. Neurologic: Peripheral neuropathy (mainly with bortezomib; dose-limiting; sensory or painful). Cardiovascular: heart failure, hypertension, arrhythmias, and acute coronary syndromes (specifically for carfilzomib)	[74,75]
BH3 mimetics	Hematologic: cytopenia. Thrombocytopenia, especially associated with navitoclax (dose-limiting). Increased infection risk. Other: tumor lysis syndrome.	[47]
MEK inhibitors	Hematologic: mild cytopenia. Dermatologic: skin rash, pruritus, alopecia, hand-foot skin reaction (palmar-plantar erythrodysesthesia). Ocular: retinal disorders	[76]
CDK4/6 inhibitors	Hematologic: cytopenia, especially neutropenia (dose-limiting). Mild gastrointestinal and hepatic toxicity.	[49,50]
mTOR inhibitors	Hematologic: mild cytopenia. Dermatologic: skin rash. Metabolic: hyperlipidemia, hyperglycemia.	[77]
HDAC	Hematologic: cytopenia, especially thrombocytopenia (dose-limiting). Gastrointestinal: nausea, vomiting, diarrhea, anorexia. Constitutional: fatigue, headache. Cardiovascular: QT prolongation.	[57,58]
DNA methyltransferase inhibitors	Hematologic: cytopenia. Increased infection risk. Gastrointestinal: Nausea, vomiting, diarrhea, constipation.	[60,62]
FLT3 inhibitors	Hematologic: cytopenia. Cardiovascular: QT prolongation (especially with quizartinib). Gastrointestinal: nausea, vomiting, diarrhea, anorexia. Hepatic: elevated liver enzymes.	[78]
Menin inhibitors	Hematologic: cytopenia. Gastrointestinal: nausea, vomiting, diarrhea, abdominal pain. Other: differentiation syndrome	[79]

## Data Availability

No new data were created or analyzed in this study. Data sharing is not applicable to this article.

## Supplementary Materials

The following supporting information can be downloaded at: https://www.mdpi.com/article/10.3390/cancers17203322/s1, Table S1: Clinicaltrial.gov research strategy.

## Abbreviations

The following abbreviations are used in this manuscript:

AYAs	Adolescents and young adults
ALL	Acute lymphoblastic leukemia
AML	Acute myeloid leukemia
B-ALL	B-cell precursor acute lymphoblastic leukemia
BFM	Berlin–Frankfurt–Münster
CAR-T	Chimeric antigen receptor T-cell therapy
CCCG	Chinese Children’s Cancer Group
CDK	Cyclin-dependent kinases
CML	Chronic myeloid leukemia
CML-CP	CML chronic phase
CML-AP	CML accelerated phase
CML-BP	CML blast phase
CNS	Central nervous system
COG	Children’s Oncology Group
CR	Complete remission
DFCI	Dana–Farber Cancer Institute
DNMTi	DNA methyltransferase inhibitor
EFS	Event-free survival
EsPhALL	European intergroup study Philadelphia chromosome-positive ALL
EMA	European Medicines Agency
EOI	End of induction
EOC	End of consolidation
ETP	Early T-cell precursor
FDA	Food and Drug Administration
HDACi	Histone deacetylase inhibitor
HEM-iSMART	International proof of concept therapeutic Stratification trial of Molecular Anomalies in Relapsed or Refractory HEMatological malignancies in children
HMAs	Hypomethylating agents
HR	High-risk
HSCT	Hematopoietic stem cell transplantation
IntReALL	I-BFM-SG international study for children and adolescents with relapsed ALL
LLy	Lymphoblastic lymphoma
MDACC	MD Anderson Cancer Center
MEK	Mitogen-activated extracellular protein kinase
MTD	Maximum tolerated dose
NGS	Next-generation sequencing
ORR	Overall response rate
OS	Overall survival
Ph+	Philadelphia chromosome-positive
Ph-like	Philadelphia chromosome-like
PK	Pharmacokinetics
PMC	Princess Máxima Center for Pediatric Oncology
RP2D	Recommended phase II dose
R/R R/I	Refractory/relapsed Refractory/intolerant
SJCRH	St. Jude Children’s Research Hospital
STAMP	Specifically targeting ABL myristoyl pocket
TACL	Therapeutic Advances in Childhood Leukemia and Lymphoma
T-ALL	T-cell acute lymphoblastic leukemia
TKI	Tyrosine kinase inhibitor
VXLD	Vincristine, dexamethasone, asparaginase, and daunorubicin regimen
VPLD	Vincristine, dexamethasone, asparaginase, and doxorubicin regimen
